# The *Pyrus sinkiangensis Yu PsLEA4* Gene Enhances the Cold Resistance of *Solanum lycopersicum*

**DOI:** 10.3390/plants14020180

**Published:** 2025-01-10

**Authors:** Xueying Yang, Wenjuan Zhao, Hui Li, Zhenxia Zhao, Jianbo Zhu, Jin Li

**Affiliations:** College of Life Sciences, Shihezi University, Shihezi 832000, China; 15299925708@163.com (X.Y.); 18449395133@163.com (W.Z.); 17343653459@126.com (H.L.); 18100948197@163.com (Z.Z.)

**Keywords:** LEA, *Pyrus sinkiangensis Yu*, low temperature, abiotic stress, transgenic tomato

## Abstract

Plants have large amounts of the late embryogenesis abundant protein (LEA) family of proteins, which is involved in osmotic regulation. The Korla Pear (*Pyrus sinkiangensis Yu*) is an uncommon pear species that thrives in Xinjiang and can survive below-freezing conditions. We found that the *PsLEA4* gene was more expressed after cold treatment by looking at the transcriptome data of the Korla Pear. In order to evaluate the biological function of the *PsLEA4* protein under low-temperature stress and its potential for use in agricultural breeding, we cloned the *PsLEA4* gene from the Korla Pear, made a plant overexpression vector, and transformed it into a tomato via Agrobacterium transformation. When exposed to low temperatures, we found that *PsLEA4* overexpression can regulate proline metabolism and antioxidant enzyme activity in tomatoes compared to wild tomatoes. Because of this, transgenic tomatoes are more resilient to cold temperatures and produce more than their wild counterparts. Thus, expressing *PsLEA4* has multiple advantages: (1) Improving frost resistance and reducing plant damage. (2) Increasing crop yield. Therefore, this study provides a theoretical basis for the role of the *PsLEA4* protein in plants’ resilience to low temperatures, as well as for its potential application in crop breeding.

## 1. Introduction

Abiotic stress factors in the environment, such as low temperature, high temperature, excessive light, drought, and salinity, are significantly increased due to climate change [1]. In nature, plants are exposed to a variety of stress conditions, and adaptation to these stresses includes a comprehensive response to individual stress and the formation of a new type of response [2]. The impact of abiotic stress factors depends on the type of stress, duration, and plant species [3]. It will bring disastrous effects to the ecosystem and social economy and bring huge losses to the global economy and agriculture. Therefore, it is crucial to find ways to increase crops’ resistance to stress in addition to safeguarding the natural environment. In this study, plant genetic engineering was used to increase tomatoes’ resilience to abiotic stress [4].

Late embryonic enrichment (LEA) proteins were first identified in the cotyledons of cotton by Dure et al. They are extremely hydrophilic, glycine-rich proteins that are widely distributed throughout the plant kingdom [5,6], which usually accumulate during the late stages of seed maturation and gradually disappear after germination. Typically, the LEA protein gene family has several members. For example, Michaela Hundertmark et al. discovered that the model plant Arabidopsis thaliana comprises 51 LEA protein members, which can be divided into eight subfamilies according to their amino acid sequences [7], namely LEA1, LEA2, LEA3, LEA4, LEA5, LEA6, Dehydrin, and SMP [7,8]. Relying on the development of whole genome sequencing technology, an overview of LEA protein gene families in several species is now available; e.g., rice (*Oryza sativa* L.) contains 34 LEA genes [9], wheat (*Triticum aestivum* L.) contains 179 LEA genes [10], chili (*Capsicum annuum* L.) contains 82 LEA genes [11], melon contains 61 LEA genes, watermelon contains 73 LEA genes [12], Chinese cabbage (*Chinese cabbage*) contains 65 LEA genes [13], rape (*Brassica napus* L.) contains 23 LEA genes [14], poplar (*Populus trichocarpa*) contains 88 LEA genes [15], lotus *(Nelumbo* spp.) contains 57 LEA genes [16], apricot (*P. armeniaca* L. *× P. sibirica* L.) identified 54 LEA genes [17], and so on.

Numerous studies have demonstrated that plants can become more resilient to abiotic stimuli such as drought, low temperatures, salt, and heavy metals when LEA protein is present. X. Guo et al. found that *SiDHN* and *SiDHNl*, two dehydroxin genes of Korla Pear (*Pyrussinkiangensis Yu*), could significantly improve the drought and cold tolerance of transgenic tobacco [18,19]. Zhao Wei et al. cloned the first group LEA (*As-g1lea*) and the third group LEA (*As-g3lea*) genes of Artemia sinica and found that their expression was up-regulated under low-temperature stress and up-regulated under high salt stress [20]. Hatanakan et al. found that the expression of *MpLEA1* in an LEA gene in a bryophyte, Geodetia, can prevent the accumulation of α-tyrosine under dehydration conditions, acting as a “molecular shield” to protect [21]. Ren Jiangling et al. found that the *PmLEA1* gene in broom millet was involved in drought resistance regulation and different hormone signaling processes [22]. Shiraku Margaret Linyerera et al. found that the overexpression of *GhLEA3* in cotton enhanced the tolerance of transgenic Arabidopsis plants to salt and drought stress [23]. Liu et al. found that *MsLEA1* recruits and protects its target proteins (SOD and Ms1770) and increases alfalfa tolerance against drought and Al stresses [24]. Guo Binhui et al. found that the overexpressed *GmLEA4_19* soybean showed a drought tolerance phenotype. These results indicated that *GmLEA4_19* plays an important role in the tolerance to drought and will contribute to the development of the soybean transgenic with enhanced drought tolerance and better yield [25]. Jing Yu et al. reported that overexpression of *OsLEA3-2* could improve drought tolerance and salt tolerance of rice [26]. Y. Liu et al. found that *ZmLEA3* was involved in responding to low-temperature stress effects in LEA protein family members of maize, which enabled Escherichia coli and transgenic yeast to have low-temperature tolerance [27], and the expression of alfalfa (*MfLEA3*) composition improved the cold tolerance and drought tolerance of transgenic tobacco [28].

“Hanhai Pear, out of Hanhai north, cold-resistant not withered” is one of the “Xijing miscellany” records that Jin dynasty Ge Hong gathered. The term “hanhai Pear” describes the Korla Pear (*Pyrus sinkiangensis Yu*) species found in the Tarim Basin of Xinjiang, which belongs to the Rosaceae, Pomindeae, and Pyrus families. It has a history spanning over two millennia and is indigenous to southern Xinjiang. With its thin skin, crispy meat, juicy, sweet, crisp, refreshing, nutrient-rich, versatile, and cold-resistant qualities, it is one of the most representative fruits in Xinjiang and has a distinct regional identity [29]. Our experimental team’s transcriptome and genome sequencing in the prior investigation revealed that *PsLEA4* gene expression was markedly elevated in response to low-temperature stress. This gives us a reliable database for exploring *PsLEA4*’s function in more detail. The *PsLEA4* protein, which is encoded in Korla Pear (*Pyrus sinkiangensis Yu*), was cloned for this research. The function of *PsLEA4* transgenic tomato in response to low-temperature stress was examined, plant overexpression vectors were created, transgenic tomato plants were produced by Agrobacterium-mediated infestation, and the gene sequence was examined using bioinformatics methods. By evaluating the morphology, traits, photosynthetic parameters, and physiological and biochemical characteristics of transgenic tomato plants, it was shown that *PsLEA4* may play an important role in plant low-temperature stress resistance.

## 2. Results

### 2.1. Analysis of the Expression Characteristics of the LEA Gene in Korla Pear Under Overwintering Conditions

**Figure 1:**
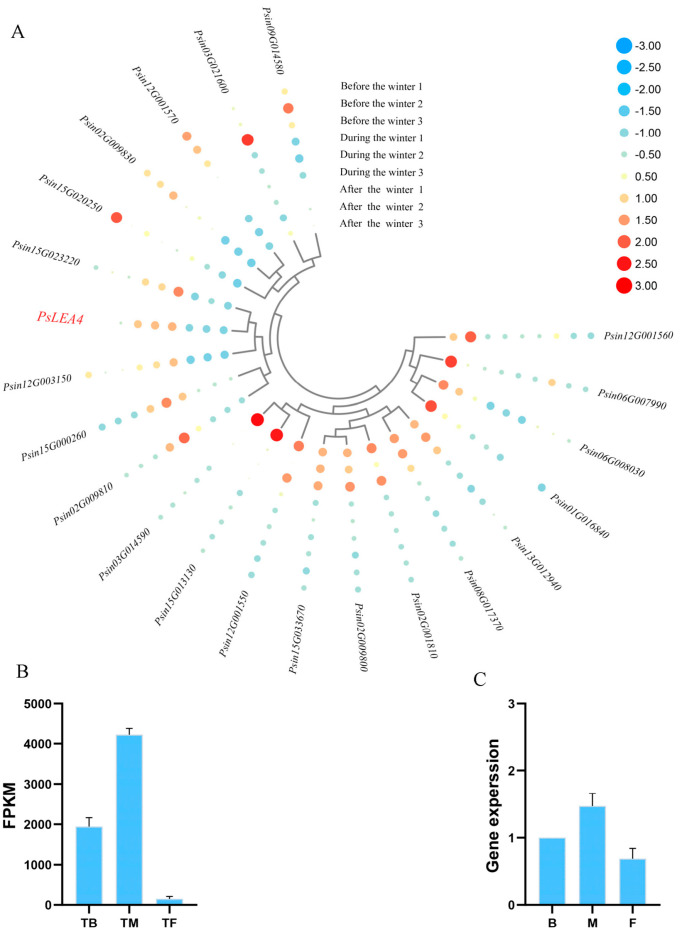
Expression of *PsLEA4* gene during overwintering. ((**A**) indicates a heat map of 21 LEA differentially expressed genes, with three replicates in each period. Red represents high expression, blue represents low expression, and the size of the circle represents the size of gene expression; (**B**) indicates the results of *PsLEA4* transcriptome sequencing; (**C**) indicates that the *PsLEA4* transcriptome sequencing results were verified by qRT-PCR).

Through the analysis of transcriptome data in three periods during the overwintering process of Korla Pear, it was found that 21 LEA genes were differentially expressed during the overwintering process. Figure 1 showed that 5 of the 21 differentially expressed genes were highly expressed in the early wintering period (TB) in October, 5 genes were highly expressed in the coldest wintering period (TM) in December, and 12 genes were highly expressed in the late wintering period (TF) in March. Among them, the expression levels of five LEA genes reached the highest in December during the coldest period of overwintering. The authors speculated that five genes of the LEA gene family in Korla fragrant Pear played a role in the resistance to low-temperature stress during overwintering. The *PsLEA4* gene was selected for fluorescence quantitative PCR. The results showed that the expression trend of *PsLEA4* gene during overwintering was consistent with transcriptome sequencing, so as to carry out subsequent experiments.

### 2.2. Bioinformatics Analysis of PsLEA4 Gene

**Figure 2:**
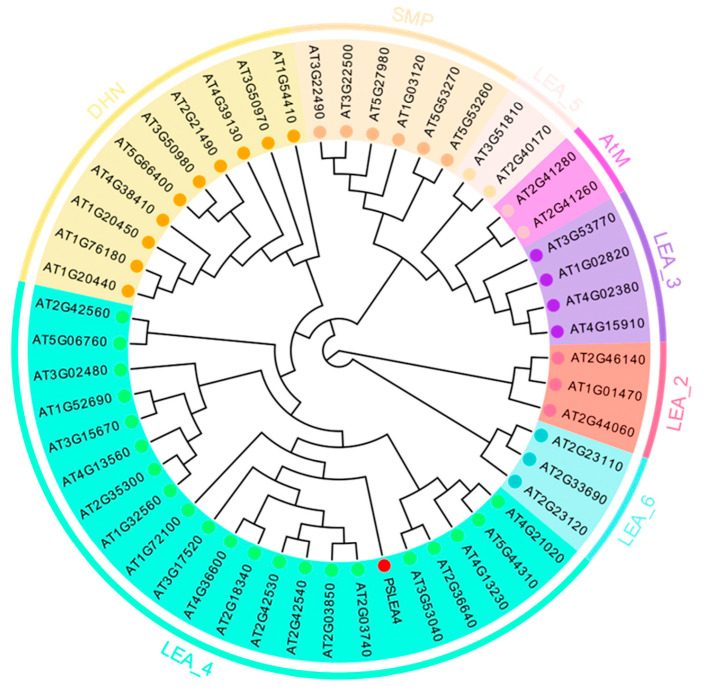
Phylogenetic tree of the *PsLEA4* gene. (The phylogenetic tree was constructed by MEGA11.0, and 1000 bootstrap repeats were established using the neighbor-joining method. Regions of different colors represent different subfamilies of LEA).

The full-length (1620 bp) sequence of the *PsLEA4* gene was cloned from Korla Pear. The sequence had an open reading frame of 1295 bp and encoded a protein of 369 amino acids. analyze the physicochemical properties of *PsLEA4*. The protein’s structural and physicochemical properties were investigated. The protein had an electrical point PI of 5.58 and was a hydrophilic transmembrane protein. 69 basic amino acids and 74 acidic amino acids are present. The protein was stable, as shown by the fat index of 46.06 and the instability index of 18.59, both of which were below 40. The *PsLEA4* gene protein’s signal peptide was predicted. The findings indicated that, with a probability of 0.37 percent, a signal peptide was present at amino acid positions 28 and 29. The protein *PsLEA4* is secreted. The protein sequences of the acquired Arabidopsis LEA family genes were analyzed and annotated using Pfam, and visualization analysis was carried out using TBtools (v2.056) [30] tools. The findings demonstrated that the *PsLEA4* protein shares conserved structural domains with the Arabidopsis LEA-4 family. The *PsLEA4* protein is a member of the LEA-4 subfamily, according to the phylogenetic tree we created using MEGA 11.0 (various colors correspond to different subfamilies) (Figure 2). Consequently, it was given the designation *PsLEA4*.

### 2.3. Morphological and Physiological Changes in Transgenic Tomato Plants Overexpressing PsLEA4 Under Low-Temperature Stress

**Figure 3:**
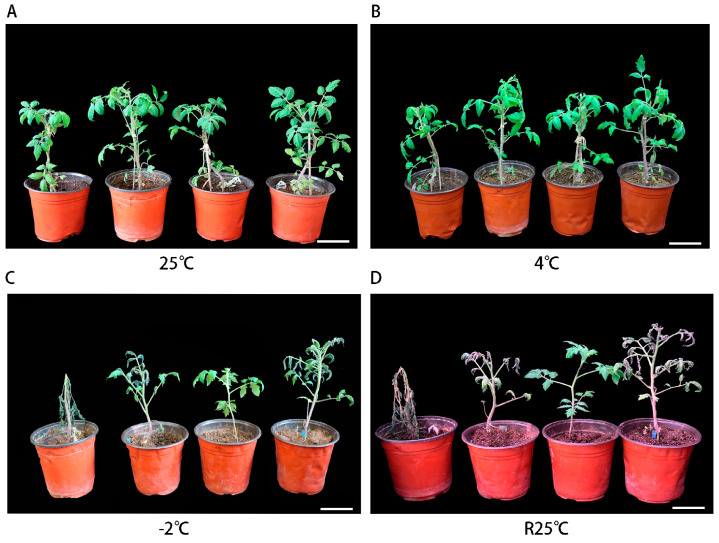
Phenotypes of wild and overexpressing *PsLEA4* transgenic tomato plants under cold stress. ((**A**) Wild and transgenic tomatoes were grown in a greenhouse at 25 °C for 6 weeks. (**B**) The growth of wild tomato and transgenic tomato in a 4 °C low-temperature incubator after 8 h. (**C**) Morphological differences between wild tomato and transgenic tomato after 6 h of low-temperature culture at −2 °C. (**D**) Morphological maps of transgenic tomatoes and wild tomatoes after 3 days of recovery under low-temperature stress at 25 °C. Bar = 7 cm).

To prove that *the PsLEA4* gene has low-temperature tolerance in tomatoes, we observed the phenotypic changes of overexpressing *PsLEA4* transgenic tomatoes and wild tomatoes by low-temperature stress treatment (25 °C, 4 °C, and −2 °C). The overexpression of *PsLEA4* transgenic tomato(Supplementary Figures S1 and S2) did not change substantially after 8 h of treatment at 4 °C, but the wild tomato’s leaves did exhibit some curling. Following a 6 h treatment period at −2 °C, the wild tomato’s leaves were badly wilted and nearly inactivated. The overexpressing *PsLEA4* transgenic tomato displayed withering and bending of its leaves. Following three days of culture at 25 °C, the leaves of the overexpressing *PsLEA4* transgenic tomato strain progressively recovered, while the wild strain’s stem was badly bent and did not recover (Figure 3).

Relative water content (RWC) and relative electrical conductivity (REC) are frequently employed as measures of plant stress tolerance. Relative water content (RWC) indicates the strength of plant metabolism [31]. Relative electrical conductivity (REC) can indicate the extent of damage to plant cell membranes [32]. The results showed that previous to treatment, the relative water content of transgenic and wild lines was comparable. As the treatment temperature was reduced, the relative water content of both wild and overexpression lines decreased; nevertheless, the relative water content of transgenic lines remained much higher than that of wild lines. The OE-2 overexpression lines had a higher relative water content than OE-1 and OE-3. As the treatment temperature dropped, the relative conductivity of both transgenic and wild lines increased. At temperatures under −2 °C, the relative conductivity of wild lines reached 93.56%, whereas OE-1 showed 55%, OE-2 showed 36%, and OE-3 showed 48%. It demonstrates that the cell membranes of wild lines were significantly damaged, with OE-2 showing the least amount of damage.

Malondialdehyde (MDA) content is a crucial metric indicating the organism’s potential antioxidant capacity [33], which indicates the rate and severity of lipid peroxidation and indirectly represents the extent of peroxidative tissue damage [34]. The results showed that with the decrease in treatment temperature, the MDA content of wild and transgenic tomatoes increased, and the increase in wild strains was more obvious. At 4 °C or −2 °C, the MDA content of WT strains was significantly higher than that of transgenic strains.

**Figure 4:**
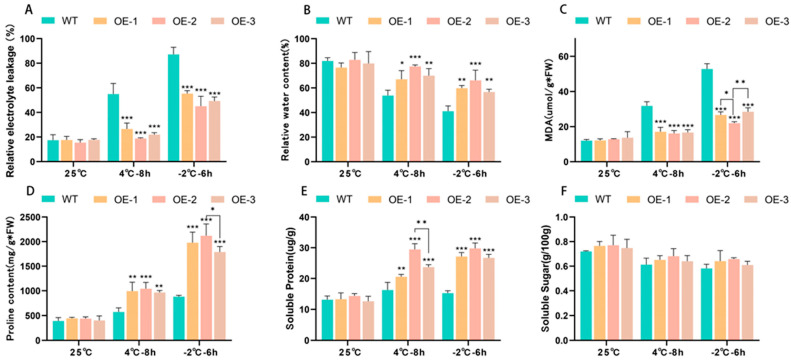
Physiological changes in wild and transgenic tomato lines overexpressing *PsLEA4* under cold stress. ((**A**) REL (%), (**B**) RWC (%), (**C**) MDA content, (**D**) pro content, (**E**) soluble sugar, (**F**) soluble protein. The data were three replicates of the average SD. The asterisk indicates that there were significant differences between wild and transgenic plants: * *p* < 0.05, ** *p* < 0.01, *** *p* < 0.001).

Soluble sugars and soluble proteins serve as crucial osmotic regulators and nutrients [35,36]. Their increase and accumulation can improve cells’ water storage capacity and protect their life substances and biofilms [37]. Proline is a constituent of plant proteins and is commonly found in free form within plants [38]. Under stress conditions (drought, salinity, cold, freezing), the content of proline in plants increased significantly. The proline level present in plants indicates their tolerance to stress to a certain degree. The results (Figure 4) indicated that a drop in temperature led to an increase in proline and soluble protein levels in both wild and transgenic tomatoes. The concentration of the three was greatest at −2 °C, with the accumulation of transgenic tomatoes markedly exceeding that of wild tomatoes. No substantial variation in soluble sugar concentration was observed between the wild and transgenic strains.

**Figure 5:**
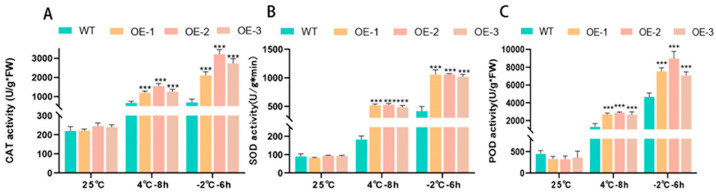
Changes in related enzyme activities in wild tomatoes and transgenic tomatoes overexpressing *PsLEA4* under cold stress. (**A**) CAT content, (**B**) SOD content, (**C**) POD content, the asterisk indicates that the difference between wild tomatoes and transgenic tomatoes was significant: *** *p* < 0.001).

Plants have an antioxidant system that eliminates the production of reactive oxygen species (ROS) [39,40], and the ability of the body’s antioxidant system to scavenge reactive oxygen species decreases under adverse conditions [41]. Plant stress tolerance can be assessed by measuring the levels of catalase (CAT), superoxide dismutase (SOD), and peroxidase (POD) [42,43,44,45,46,47]. The results (Figure 5) showed that the accumulation of CAT, SOD, and POD increased gradually with the decrease in treatment temperature, and the accumulation of transgenic plants was significantly higher than that of wild plants. At −2 °C, transgenic tomatoes’ CAT, SOD, and POD were 4.11, 2.48, and 1.68 times higher than those of wild tomatoes, respectively. According to this, transgenic tomatoes are better than natural tomatoes at scavenging reactive oxygen species (ROS).

### 2.4. Evaluation of Tomato Overexpressing the PsLEA4 Gene’s Fruit Yield and Photosynthetic Potential

**Figure 6:**
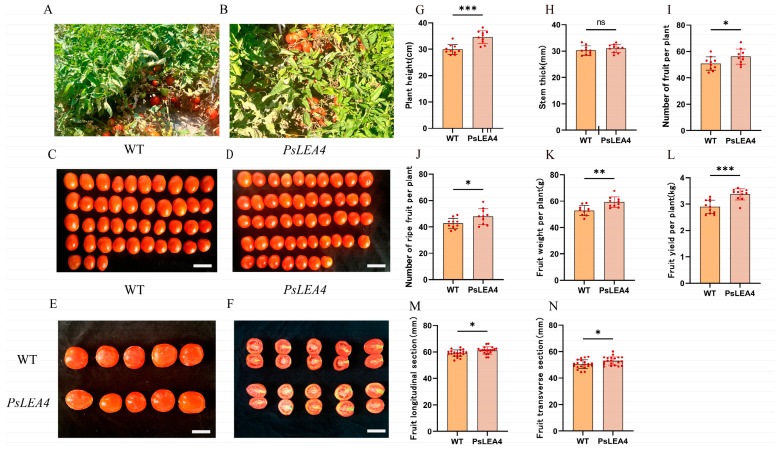
Analysis of yield and agronomic traits of *PsLEA4*. (**A**,**B**) Field status of 16-week-old wild tomato plants and *PsLEA4* transgenic tomato plants; (**C**,**D**) statistics of mature fruits of WT plants and *PsLEA4* transgenic plants. (**E**,**F**) longitudinal section of wild fruit and *PsLEA4* transgenic fruit; (**G**,**H**) comparison of plant height and stem diameter between wild tomato plants and *PsLEA4* transgenic lines; (**I**–**L**) yield analysis of wild tomato plants and *PsLEA4* transgenic plants; (**M**,**N**) longitudinal comparisons of wild tomato plant and *PsLEA4* transgenic plant fruit cross-sections. Bar = 50 mm. The asterisk indicates a statistically significant difference (* *p* < 0.05, ** *p* < 0.01, *** *p* < 0.001, ns means no significant difference).

**Figure 7:**
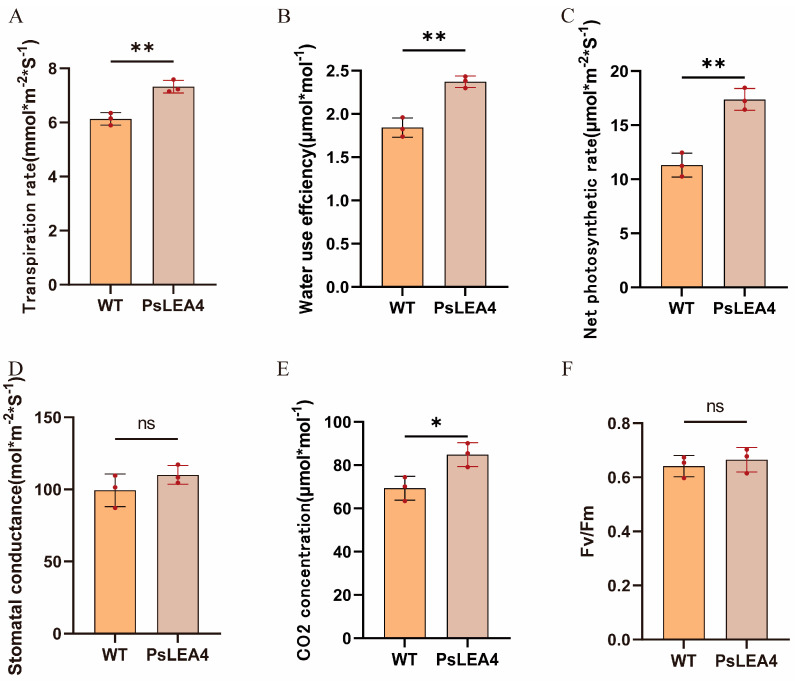
Measurement of light and capacity of wild tomato plants and transgenic tomato plants. (**A**) Transpiration rate, (**B**) water utilization, (**C**) net photosynthetic utilization, (**D**) stomatal conductance, (**E**) intercellular carbon dioxide concentration, and (**F**) Fv/Fm. (* *p* < 0.05, ** *p* < 0.01, ns means no significant difference).

The results showed that (Figure 6): At 90 days of normal growth, by measuring the length of the tomatoes, the transgenic tomatoes were slightly higher than WTs, and there was no difference in stem diameter between the two sides. The number of fruits per plant of transgenic tomato plants was higher than that of WT plants. Moreover, the number of mature fruits per fruit of transgenic tomatoes was higher than that of WTs. In the measurement of fruit yield, the average yield and average fruit weight of transgenic plants were also significantly higher than those of WT plants. Moreover, we measured the light and capacity of transgenic tomato plants and wild tomatoes (Figure 7). The transpiration rate of wild plants was 6.133 (mmol·m^−2^·S^−1^), and the transpiration rate of transgenic tomato plants was 7.325 (mmol·m^−2^·S^−1^). The net photosynthetic rate of wild plants was 11.314 (μmol·m^−2^·S^−1^), and the net photosynthetic rate of transgenic tomato plants was 17.37967 (μmol·m^−2^·S^−1^). The Fv/Fm of wild plants was 0.6419, and the Fv/Fm of transgenic tomato plants was 0.6652.

## 3. Discussion

Global climate change is one of the major constraints limiting plant growth, production, and sustainability worldwide [48]. However, climate change, combined with increased frequency and intensity of extreme weather events such as droughts, heat waves, floods, and cold dangers, threatens agri-food systems around the world [49]. To effectively tackle the challenges of modern agriculture, it is critical to develop novel crops that are resistant or tolerant to environmental pressures. We established the *PsLEA4* gene of Korla Pear through transgenic means from the perspective of genetic engineering to improve the cold-resistant ability of tomatoes. The yield of the tomato is better than that of the wild tomato, which provides the theoretical basis of tomato agricultural production.

Plant growth and development are significantly impacted by abiotic stress [50,51,52,53,54]. Under stress, plants are susceptible to harm that can impede their growth and development and even cause them to die. Stress causes plant cells to lose their homeostasis, which leads to cell state instability [55]. LEA protein is an IDPS protein [56], which is induced by drought, freezing, salt, and other stresses in plants, affecting plant growth and development [57]. Although the LEA4 protein’s C-terminus is not well conserved, its N-terminus contains a hydrophilic conserved region made up of [58,59,60,61,62,63,64,65,66,67,68] amino acids. The N-terminus of these proteins will assume an α-helix shape in the event of macromolecular crowding or water deficit, giving the LEA4 protein the ability to provide protection in vitro [69].

The cultivation of stress-resilient crops with enhanced yield stability is the most effective strategy for overcoming multiple and fluctuating environmental cues. Natural genetic variation in crops, genetic engineering, chemical interventions, and microbial stimulation [70]. Previous studies have shown that overexpression of LEA4-4 in alfalfa improves salt tolerance, drought tolerance, and antioxidant resistance in transgenic Arabidopsis thaliana [71]; *CiLEA4* basal expression and nutritional development level of chicory varieties are more tolerant to drought stress conditions [72]; *BnLEA4-1* in kale is overexpressed in Escherichia coli and transgenic Arabidopsis thaliana plants: Overexpression of *BnLEA4-1* cDNA in E. coli was salt- and temperature-tolerant, and transgenic Arabidopsis thaliana plants with *BnLEA4-1* showed tolerance to salt and drought stress [73]; rice (*Oryza sativa* L.) *LEA4-5* was analyzed as a novel salt-tolerance-responsive gene by transcriptome data [74]; the LEA4 gene, as a gene related to the regulation of senescence in apple seeds when subjected to NO treatment, would have an effect on seed senescence [75]. The above results indicate that LEA4 protein plays an important role in abiotic stress tolerance at the vegetative stage of plant development.

In this study, we plotted heat maps to analyze the differentially expressed genes of LEA in transcriptome data and found that the expression of the *PsLEA4* gene was the highest in the coldest period of overwintering. The expression of the *PsLEA4* gene was verified by QRT-PCR, and the results were consistent with the transcriptome data. We cloned and analyzed the *PsLEA4* gene of Korla Pear, which was rapidly up-regulated under cold stress. The *PsLEA4* protein overexpression vector for plants was created. *PsLEA4*’s impact on plant growth and development in the face of abiotic stress was examined. Our experimental findings showed that transgenic tomatoes’ resistance to low-temperature stress was enhanced by *PsLEA4* overexpression. When exposed to stress at 4 °C and −2 °C, transgenic tomato plants overexpressing *PsLEA4* demonstrated resilience to low temperatures. We assessed the resistance of transgenic tomato plants overexpressing *PsLEA4* in a comprehensive manner using the outward phenotypic observation as well as the internal physiological and biochemical alterations of wild and transgenic tomatoes.

Studies have shown that plants have problems such as water loss and cytoplasmic membrane damage under low-temperature stress. Plant water status and osmotic adjustment are measured using the relative water content (RWC), which represents the plants’ ability to retain water and metabolic strength [31]. In this study, under low-temperature settings, the RWC of transgenic tomatoes overexpressing *PsLEA4* was considerably higher than that of WTs. The ability of transgenic *PsLEA4* tomato plants to sustain water homeostasis and metabolic processes in low-temperature environments was demonstrated. To a certain degree, the amount of proline in plants indicates how resistant they are to stress. Because proline is hydrophilic, it can lower the freezing point and stop cell dehydration by stabilizing the metabolic process of plant tissue and protoplast colloid [76]. Proline levels in plant tissues rise in colder climates, which may help plants withstand colder temperatures. LEA-encoded soluble proteins aid in preserving membrane integrity and protein structure while averting excessive cytoplasmic dehydration. In plants, soluble protein is a crucial component for osmotic adjustment and nutrition. Its build-up can protect plants and increase cells’ ability to retain water [77]. In the current result, transgenic tomato plants overexpressing *PsLEA4* had larger levels of soluble protein and proline than natural tomato plants grown at low temperatures. Transgenic tomato plants overexpressing *PsLEA4* had a soluble protein content 1.83 times higher than wild tomato plants and a proline content 2.221 times higher than wild plants, particularly at −2 °C. The malondialdehyde (MDA) level and relative electrical conductivity (REL) can typically indicate the extent of cell membrane damage in plants [78]. In our study, the degree of damage to transgenic plants was much lower than that of wild plants, and the relative conductivity and malondialdehyde of wild plants were significantly greater than those of overexpressing *PsLEA4* transgenic plants as the stress level increased. This suggests that the transgenic plants can increase their capacity to store water, preserve their biofilm, increase their resistance to low temperatures, maintain the equilibrium of osmotic pressure both inside and outside of cells, and accumulate soluble protein and proline content more rapidly following stress [79]. ROS can induce cell damage through protein degradation, enzyme inactivation, and genetic changes and interfere with various metabolically important pathways. The potential role of its molecular mechanism during abiotic stress is very important for the methods of regulating plant growth and metabolism under stress conditions [58,80]; we found that CAT, POD, and SOD of transgenic tomato plants were significantly higher than those of wild tomatoes under low-temperature stress. Especially at −2 °C, it was 4.11 times, 1.68 times, and 2.48 times that of wild tomato plants, respectively, indicating that overexpressing *PsLEA4* transgenic tomato plants had a stronger ability to scavenge ROS than wild plants. Lastly, we compared the photosynthetic potential and agronomic characteristics of transgenic and wild tomato plants.

Tomato is one of the main economic vegetable crops in the world today; the study of tomato yield enhancement is significant for tomato agricultural cultivation [59,60,61,62]. However, high yield and stress tolerance are often difficult to achieve at the same time, which has also been a difficult point of attack for so many years of breeding research. The role of LEA proteins in controlling the growth and development of tomato fruits is currently less well reported.

In this study, we conducted an experimental field trial to compare the agronomic traits of wild tomatoes, *PsLEA4* gene overexpressing tomatoes, WT, and transgenic tomatoes under field cultivation conditions in various aspects. The findings demonstrated that transgenic tomato plants with overexpression produced better results than wild tomato plants. During the same time period, transgenic tomato plants produced more mature fruits per plant than wild tomato plants, and their fruit weight and longitudinal section were as high as those of wild tomato plants. These findings suggest that LEA protein may have a role in plant growth and development in addition to its association with plant stress resistance. The intercellular CO_2_ net photosynthetic rate, transpiration rate, and stomatal conductance of transgenic tomato plants were all noticeably higher than those of wild tomato plants when determining the photosynthetic capacity of plants. The Fv/Fm ratios of transgenic and wild tomato plants did not differ significantly. According to the aforementioned findings, the *PsLEA4* protein regulates stomatal conductance and intercellular CO_2_ concentration, which enhances photosynthetic rate and water usage efficiency in plants. Consequently, plants are better able to retain water, which increases the amount of water that plants absorb through transpiration. The yield of transgenic tomato plants is marginally higher than that of WT plants for the same reasons.

In summary, transgenic tomatoes that overexpress *PsLEA4* are stronger in resistance to cold temperatures than wild tomatoes. We think that the *PsLEA4* protein may be able to detect the harm that low temperatures cause to transgenic tomato plants, as well as the damage that dehydration and elevated reactive oxygen species (ROS) cause to organelles and plants. *PsLEA4* is responsible for maintaining the stability of the cell membrane structure. It protects the cell membrane and organelles by attaching itself to the membrane when it is dehydrated and providing a protective matrix. In addition, it might function as a “molecular chaperone” to preserve the integrity of the cell–matrix and membrane, as well as the protein structure and enzyme activity [63]. Additionally, the *PsLEA4* protein can enhance the plant’s photosynthetic rate and water use efficiency by controlling stomatal conductance and intercellular CO_2_ concentration. The yield of transgenic tomatoes that overexpress *PsLEA4* is marginally higher than that of wild tomato plants. Therefore, our findings indicate that *PsLEA4* has a promising future application in cold resistance breeding.

## 4. Materials and Methods

### 4.1. Plant Materials

The Korla Pear (National Horticultural Germplasm Resource Bank: ZGSPY180) cultivated in the Pear Variety Resource Conservation Park of the Institute of Agricultural Science, Korla City in Xinjiang (Chinese: Korla). The tomato (*Solanum lycopersicum* L.) variety used in axin 87-5′ wild tomato seeds is supplied by Yaxin Seed Co., Ltd. (Shihezi, China).

### 4.2. Analysis of Differentially Expressed Genes of LEA in Korla Fragrant Pear by Overwintering Transfer Group

According to the previous transcriptome sequence data of the 1-year-old branch phloem of Korla Pear [64], the expression analysis of the LEA gene family of Korla Pear was carried out. The sampling time of the overwintering transcriptome of Korla Fragrant Pear was the early overwintering period (TB) in mid-October 2019, the coldest overwintering period (TM) in mid-January 2020, and the late overwintering period (TF) in mid-March 2020. According to the genome ID number, the gene expression information of the LEA gene during the overwintering process was extracted from the transcriptome data, the differentially expressed genes were screened, and the heat map was drawn using TBtools.

The author selected the differentially expressed LEA gene during the overwintering process for RT-PCR verification. The primer design is shown in (Supplementary Sequence S1). The total PCR system was 20 μL. Each tube was added with 2 μL RNA template, upstream and downstream primers were 0.5 μL and 10 μL SYBR fluorescent dye, and finally supplemented with deionized water to 20 μL. PsGADPH was selected as the internal reference gene and calculated using the 2^−ΔΔCt^ method.

### 4.3. Bioinformatics Analysis of PsLEA4 Gene

ProtParam (https://web.expasy.org/protparam/ assessed on 3 November 2024), ProtScale (https://web.expasy.org/protscale/ assessed on 3 November 2024) and SignalP-6.0 (https://services.healthtech.dtu.dk/services/SignalP-6.0/ assessed on 3 November 2024), and were among the methods used to investigate the physicochemical characteristics and structure of the *PsLEA4* gene. All sequences of LEA proteins were downloaded from the Arabidopsis thaliana database, and the homology of the *PsLEA4* gene was identified using BlastP from NCBI (Supplementary Sequences S3 and S4). DNAMAN software was used for sequence analysis, ClustalW (version 2.0) [65] for multiple comparisons, and finally MEGA-11.0 [65] and Itol (https://itol.embl.de/ assessed on 29 November 2024) were used to complete phylogenetic analysis and visualization.

### 4.4. Cloning of PsLEA4 and Construction of Plant Expression Vector

The RNAisoPlus kit (TaKaRa, Biomedical Technology (Beijing) Co., Ltd., Beijing, China) was used to extract total RNA from the leaves of the Korla Pear, and the reverse transcription kit (TaKaRa) was used to create cDNA in accordance with the production instructions. Primer 6.0 was Utilized to generate particular primers based on the *PsLEA4* gene sequence. KpnI and BamH I’s restriction sites were supplemented with *PsLEA4* (Supplementary Sequence S2). The *PsLEA4* gene fragment was obtained by PCR amplification using the cDNA as a template.

### 4.5. PsLEA4 Transgenic Tomato Plant Transformation

To create sterile tomato seedlings, tomato seeds of the variety “Yaxin 87-5” were sterilized with 75% ethanol and 5% sodium hypochlorite for 8–10 min. The seeds were then sown on 1/2 MS medium, dark cultured for two days, and light cultured for seven days. After the sterile tomato seedling’s second true leaf emerged, the hypocotyl was cut into stem segments measuring around 1 cm, placed on pre-culture medium for two days of dark culture, and the *PsLEA4* gene was converted into tomato stem segments using the Agrobacterium-mediated transformation. The *PsLEA4* gene was inserted into tomato stem segments, which were then grown for 50–60 days in MS medium containing the screening hormone kanamycin. The MS medium was amended every 15–20 days. After the healing wound divided into seedlings, the seedlings were transferred into rooting medium and allowed to form roots for 20 days before being planted in culture pots for management and cultivation. 25–28 °C, 65–70% relative humidity, 7000 lx light intensity, and a 16–8 h light/dark cycle were the culture conditions.

The Plant Genome Extraction Kit and the RNAiso Plus Kit (Takara, Biomedical Technology (Beijing) Co., Ltd., Beijing, China) were used to extract the tomato’s total RNA. The recombinant plasmid pCAMBIA2300-*PsLEA4* served as the positive control, wild tomato (WT) served as the negative control, and cDNA served as the template. The following amplification program was used to identify the transformed tomato T0 generation: 95 °C for 5 min, 95 °C for 30 s, 64 °C for 30 s, 72 °C for 1 min, 20 s, 35 cycles, and finally, 72 °C for 7 min. In order to produce T1 generation seeds for further experimental research, the produced transgenic tomato was kept in culture.

### 4.6. Measurement of Physiological Indices Related to Stress Tolerance

In the process of measuring the associated physiological and biochemical indices, the leaves of both wild and transgenic tomatoes were weighed both before and after the stress treatment. In the preliminary study, we treated the wild and transgenic tomatoes with stress at 4 °C for 8 h and −2 °C for 6 h. The relative water content (RWC) of the leaves was determined by the weighing method. The fresh weight of tomato leaves was determined following treatment. To measure the saturated fresh weight of the leaves, they were soaked in distilled water for ten hours. To obtain the dry weight, the leaves were dried for 12 h at 60 °C in a hot oven. RWC is equal to the ratio of fresh weight to dry weight/saturated fresh weight to dry weight × 100%. The EC215 conductivity meter (Markson Science Inc., Henderson, NC, USA) was used to measure the relative conductivity [66]. A conductivity meter was used to test the conductivity L1 after the leaves were immersed in distilled water for 12 h. After the leaves were cooked for half an hour, the conductivity of L2 was measured once more. To calculate REL, use the formula REL (%) = L1/L2 × 100%. The sulfosalicylic acid technique was used to determine the proline content [67]. The mixture was then heated for 15 min and filtered. After 30 min in a boiling water bath, the filtrate was mixed with 2 milliliters of 2.5% acidic ninhydrin solution and 2 milliliters of glacial acetic acid. Four milliliters of toluene were added, agitated thoroughly, and layered after cooling. The top solution was then moved to a fresh 10 mL centrifuge tube and spun at 3000 r/min for 5 min. The absorbance value and the standard curve that was created using the prior proline were compared. We used the thiobarbituric acid (TBA) method to measure the amount of malondialdehyde (MDA) [68]. Following the 0.1 g weight measurement, 400 μL of liquid nitrogen grinding was added to the fresh leaves, which were then centrifuged for 15 min at 6000 rpm. On a fresh centrifuge tube, 400 μL of the supernatant was poured, 0.5% TBA was dissolved in 5% trichloroacetic acid, and 1 mL TCA was added. The absorbance value was measured at 600 nm after it had been sufficiently mixed, cooled to room temperature on ice, and centrifuged at 5000 r/min for 15 min. It had then been submerged in boiling water for 30 min. MDA content was computed as C (mol/L) = 6.45 (A532 − A600) − 0.56 (A450) at 532 nm and 450 nm [81]. The absorbance was measured by a UV1901PC ultraviolet spectrophotometer (Shanghai Ao’xi Scientific Instrument Co., Ltd., Shanghai, China). Soluble protein content was analyzed using the Coomassie brilliant blue method [82,83]. We weighed 0.1 g tomato leaves, added 5 mL of buffer, ground it into homogenate, centrifuged at 3000 r/min for 10 min, and took the supernatant for use. The sample extract (1.0 mL) was added to 5 mL of Coomassie brilliant blue reagent and shaken well. After 2 min, the absorbance was measured at 593 nm, and the protein content was detected using the glucose standard curve. Soluble sugar was determined by anthrone colorimetry [84]. We mixed 10 mL of distilled water with 0.1 g of fresh leaves, boiled for 30 min, let cool to room temperature, and then fixed to 100 mL. We measured the absorbance at 625 nm after adding 1 mL of the solution to 5 mL of 0.2% anthrone solution. The glucose standard curve was compared to the absorbance value.

### 4.7. Applicability Evaluation of Transgenic Tomato

Wild tomatoes and transgenic tomatoes were planted in the experimental field of Shihezi University. In order to explore the function and potential uses of *PsLEA4* protein in agriculture, each tomato plant’s height, stem diameter, fruit count, weight, and cross-section were compared between wild and transgenic tomatoes. During the cultivation, standard field management was practiced. Measurements of the plants’ photosynthetic capacity included stomatal conductance, intercellular CO_2_ concentration, transpiration rate, and net photosynthetic rate. The ratio of net photosynthetic rate to transpiration rate was used to determine water usage efficiency (WUE). For the precise determination procedure, please consult the GSF-3000 instructions. The transgenic plants’ leaves were measured in the same area as the wild plants. Every parameter was measured from 9:30 to 11:00.

### 4.8. Statistical Analysis

All data were initially collated using Excel 2021. Adopted Statistical analysis was performed using SPSS 23.0 and GraphPad Prism 9.5 software. Duncan’s multiple comparison test was used to determine wild type and transgenic base. There are significant differences between the lines. Significant difference level: * *p* < 0.05. The difference is significant. ** *p* < 0.01 indicates that the difference is extremely significant. All the error bars in the figure represent the standard deviation from the mean.

## Data Availability

The original contributions presented in this study are included in the article/supplementary material. Further inquiries can be directed to the corresponding authors.

## Supplementary Materials

The following supporting information can be downloaded at: https://www.mdpi.com/article/10.3390/plants14020180/s1, Figure S1. Identification of overexpression of *PsLEA4* Solanum lycopersicum at the DNA level. Figure S2. Identification of overexpression of *PsLEA4* Solanum lycopersicum at the RNA level; 1–6 are transgenic type; Sequence S1. *PsLEA4* primers; Sequence S2. RT-PCR *PsLEA4* primers; Sequence S3. *PsLEA4* CDS sequence; Sequence S4. *PsLEA4* Protein sequence.
