# Supplementary material for: The Pyrus sinkiangensis Yu PsLEA4 Gene Enhances the Cold Resistance of Solanum lycopersicum
Source: Plants (Basel). 2025 Jan 10;14(2):180. doi: 10.3390/plants14020180 (PMC11769121; doi:10.3390/plants14020180)
Supplement: Supplementary file 1 [file plants-14-00180-s001.zip › plants-3371183-supplementary.pdf]

Supplementary Data

Overexpression of *PSLEA4* Identification of transgenic strains

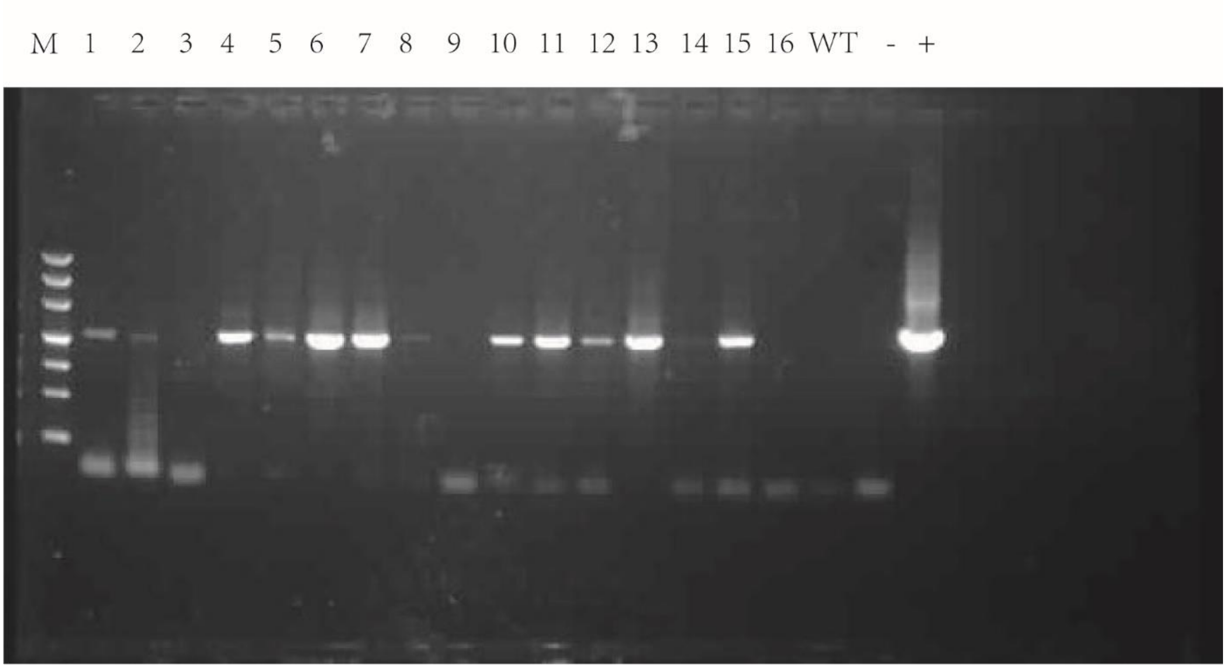

Figure S1. Identification of overexpression of PSLEA4 Solanum lycopersicum at the DNA level.1-16 are transgenic type (The numbers in the figure represent only the Spotting holes numbers, not the transgenic strain. WT- is wild-type control, - is negative control, + is positive control, M is Maker .

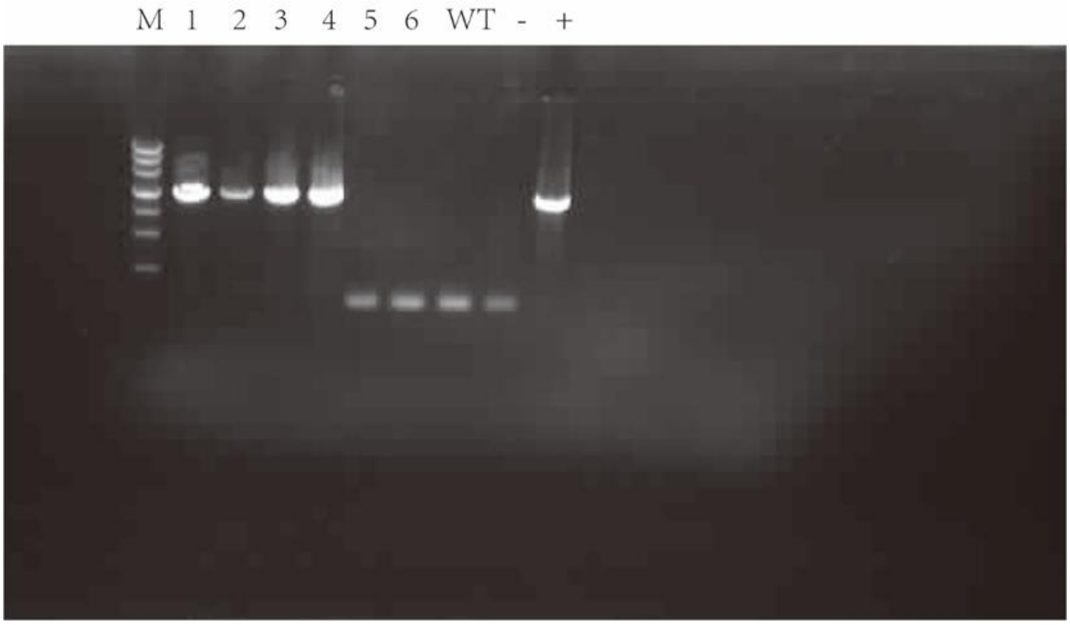

Figure S2. Identification of overexpression of PSLEA4 Solanum lycopersicum at the RNA level; 1-6 are transgenic type. WT- is wild-type control, - is negative control, + is positive control, M is Maker.）

Sequence S1. PsLEA4 primers.

PSLEA4-F: GGATCCATGGCCCAATGCGGAGGA

PSLEA4-R: CGCGGATCCGCCGACTCGTAACATCAGAC

Sequence S2. RT-PCR *PsLEA4* primers.

PSLEA4-F:TGGGCTGAAGCAGGATGATG
PSLEA4-R: TTTGGCCGATTTAGTGGCCT

Sequence S3. PSLEA4 CDS sequence.

ATGGGTGCAAAGCAACCTTACAAACTACTTCTTGCCATT-
GCGGTGGCGGCGGTGGTGGTGATGCTTGCAACCTTATGCCGCGGCTCCAGCGTGG
GGCATACGCCGTCTGCGAACGAGGAAGTTATTGATGAGTTTGTGGAG-
TCTAAGGACCAG-
TCAAGGCAGACGGAAGACGCGAGGGCGGCGGAGAAGGCGAGAGAGGGTAAAG
AAGGGTCGGAGTCGTGGACGGAGTGGGCTAAAGAGAAAATCACCGGAGGACTT-
GGGCTGAA-
GCAGGATGATGAGAATTATTTGAAGGATAGTGCTAAGAAGGCTTCTGATGCTACT
TATGACACTGCTTCTGGTTAGTCACTACTGTTTTTTAAATTATTTCCTACATAATTT-
GTGATGGAATGTGAAATTTTCTGCTTTATTTTGGATCAATTTTTAAATTTTGTCCAG
GAACTGGTTTTTTTTTTTTTTTTTCTTCAATGGGATACCGTTTGAGGATTTTACATTT-
GTACTGTGTTTCTTAAGTGTTTTCAATATGAACTCCAACATGGTGACGAAGAAATG
TGTAGAGATAAATTGATTAGAAATGGATGGACATTGGATAGACAATAACTGAG-
TGGCAC-
GAAACCATCACTTTTATATGATTTGTGTCCTTCGAAATGTATTTTTAATTGAAAATT
TGTAACGGTTTTATTTATTCGAATTAAAGTTTGTAAGTAGTTC-
TATTTATTCATATTTCCCACGAAACAGCGAAATGTTGAAATGTGGTGACCTAGACA
AAACATATTAGAGTGAAGTTAGTAATGTATTGTTAAGAATGATATATCTATTTA-
GAATTAATGTCTTACGTACTTTAAAAATTCAGGGGCTGGTGAGTATAGCACAGAA
AAGGCCAGAAACATTAAAGGTACGGCTGCAGAGAAATCTGGAGAGATGAAA-
GAAACAGCCGCCGAGAAGGCCGGCAAGACGATGGATGCAGCAATGGAGAAGGT
GTATGAGGCCACTAAATCGGCCAAAGACATGACGTACGATGCTGCTAATGCCG-
CAAAGGA-
GAAGGCCTACGAGGCCACTAATGCGGCTTATGAGACCACAAAGTCAGCAAAAG
ACAGGGCTTATGAGGCTACGAAGGAAAAGACTTATGATACTG-
CAGAGGCAGCGAAGGAAAAGGCTTATGAGGCGGCGAAGGCGGCGAAGGAAAA
GACTTATGGTACGGCGGGTGCAGCTAAGGA-
GAAGGCTTATGAGGCCACAAAGGAGGCGAC-
GGACAAGACTTCTGATACGGCGGGTGCAGCGAAGGAGAAGGCATACGAGGCCA
CAAAGGCGGCAAAAGACAAGACATATCAAACCAAGAATGCAGCTGAGGAGGC-
TACAAAGGCGGCGAAAGACAAAACGTATGAGACGAAGAACGCAGCTGAGGAG
ACGGCCCGAATAACAGCAGAGAAAGCGAATGAGGCTACCGGTTACGCGGCAGA-
GAAAGCAA-
GAGAAGCGAAAGAAAAAGCAGCGCAAACGGCAGAGGAGGTGAAGAACAAGG
CTTATGAGAAAGCAGAGGAGACTAAAGAGGCAAAGGAGAAGGCAAAGGA-
GAAGTCCCAGAA-
GATTAAGGACGAAGTTGCTGGCCGCGGCCGTGACGAGGAGCTCTGA

Sequence S4. *PSLEA4* Protein sequence.

MGAKQPYKLLLAIAVAAVVVMLATLCRGSSVGHTP-
SANEEVIDEFVESKDQSRQTE-
DARAAEKAREGKEGSESWTEWAKEKITGGLGLKQDDENYLKDSAKKASDATYDTAS
GAGEYSTEKARNIKGTAAEKSGEMKETAAEKAGKTMDAAMEKVYEAT-
KSAKDMTYDAA-
NAAKEKAYEATNAAYETTKSAKDRAYEATKEKTYDTAEAAKEKAYEAAKAAKEKTY
GTAGAAKEKAYEATKEATDKTSDTAGAAKEKAYEATKAAKDKTYQTKNAAEEAT-

KAAKDK-
TYETKNAAEETARITAEKANEATGYAAEKAREAKEKAAQTAEEVKNKAYEKAEETKE
AKEKAKEKSQKIKDEVAGRGRDEEL*
